# 

**Published:** 1958-10

**Authors:** 


					yi
ME^'1
THE
MEDICAL JOURNAL
OF THE
SOUTH-WEST
Incorporating
The Bristol Medico-Chirurgical Journal
October 195$
VOL. 73 (iv). No. 270 Price 4/-

				

